# Role of IL3RA in a Family with Lumbar Spinal Stenosis

**DOI:** 10.3390/ijms252010915

**Published:** 2024-10-10

**Authors:** Kai-Ming Liu, Chi-Fan Yang, Weng-Siong H’ng, Hui-Ping Chuang, Eunice Han Xian Khor, Pei-Chun Tsai, Vivia Khosasih, Liang-Suei Lu, Erh-Chan Yeh, Wan-Jia Lin, Feng-Jen Hsieh, Chien-Hsiun Chen, Shiuh-Lin Hwang, Jer-Yuarn Wu

**Affiliations:** 1 National Center for Genome Medicine, Institute of Biomedical Sciences, Academia Sinica, Taipei 115, Taiwan; gogila@ibms.sinica.edu.tw (K.-M.L.); cf.yang@microbio.sh.cn (C.-F.Y.); ws_hng@yahoo.com (W.-S.H.); eunice@ibms.sinica.edu.tw (E.H.X.K.); poipeiun@ibms.sinica.edu.tw (P.-C.T.); viviakhosasih@gmail.com (V.K.); liangsuei@gmail.com (L.-S.L.); ecyeh@ibms.sinica.edu.tw (E.-C.Y.); wanjlin@ibms.sinica.edu.tw (W.-J.L.); fred92517@gmail.com (F.-J.H.); chchen@ibms.sinica.edu.tw (C.-H.C.); 2 Resource Center for Translational Medicine, Institute of Biomedical Sciences, Academia Sinica, Taipei 115, Taiwan; ping@ibms.sinica.edu.tw; 3 Chi Hsien Spine Hospital, Kaohsiung 800, Taiwan

**Keywords:** lumbar spinal stenosis, IL3RA, IVD degeneration, estrogen receptor, MMP, collagen

## Abstract

Lumbar spinal stenosis (LSS) is a degenerative spinal condition characterized by the narrowing of the spinal canal, resulting in low back pain (LBP) and limited leg mobility. Twin and family studies have suggested that genetics contributes to disease progression. However, the genetic causes of familial LSS remain unclear. We performed whole-exome and direct sequencing on seven female patients from a Han Chinese family with LBP, among whom four developed LSS. Based on our genetic findings, we performed gene knockdown studies in human chondrocytes to study possible pathological mechanisms underlying LSS. We found a novel nonsense mutation, c.417C > G (NM_002183, p.Y139X), in *IL3RA*, shared by all the LBP/LSS cases. Knockdown of *IL3RA* led to a reduction in the total collagen content of 81.6% in female chondrocytes and 21% in male chondrocytes. The expression of *MMP-1*, *-3*, and/or *-10* significantly increased, with a more pronounced effect observed in females than in males. Furthermore, *EsRb* expression significantly decreased following *IL3RA* knockdown. Moreover, the knockdown of *EsRb* resulted in increased *MMP-1* and *-10* expression in chondrocytes from females. We speculate that *IL3RA* deficiency could lead to a reduction in collagen content and intervertebral disk (IVD) strength, particularly in females, thereby accelerating IVD degeneration and promoting LSS occurrence. Our results illustrate, for the first time, the association between *IL3RA* and estrogen receptor beta, highlighting their importance and impact on MMPs and collagen in degenerative spines in women.

## 1. Introduction

Lumbar spinal stenosis (LSS) is characterized by the narrowing of the spinal canal, which results in symptoms of nerve compression, such as low back pain (LBP), neurogenic claudication, numbness, weakness, heaviness, and loss of balance in the legs [1,2,3]. Spinal stenosis can occur as a result of a congenital smaller spinal canal, acquired physical damage, aging, and degenerative issues. A meta-analysis revealed that the pooled prevalence of clinical symptoms of LSS in the general population was 11% (mean age 62 years, 56% female), and 25 and 29% in patient populations under primary (mean age 69 years, 55% female) and secondary (mean age 58 years, 51% female) care, respectively [4]. The treatment strategy for LSS depends on its underlying cause. Although stenosis caused by a herniated disk can be treated with physical therapy and exercise [5], surgery is required in cases of severe symptomatic LSS, such as large overgrown bony protrusions on the disk.

Currently, the pathological mechanisms underlying degenerative LSS are not fully understood. However, the possible underlying mechanisms have been proposed [1,6]. Intervertebral disk (IVD) degeneration is one of the most common causes of LSS in adults. Degradation of the IVD matrix due to the activation of matrix metalloproteinases (MMPs) is a critical step in IVD degeneration. MMPs belong to a superfamily of zinc-dependent endopeptidases capable of degrading various components of the extracellular matrix for tissue remodeling during various biological processes [7]. Previous studies have demonstrated that MMPs, such as MMP-1, -2, -3, -7, -8, -9, -10, -11, -12, -13, and -14, are highly linked to IVD degeneration or lumbar disk herniation in humans [8,9,10,11,12]. IVD mainly consists of collagen type I and II matrices and small amounts of types III, V, VI, IX, and XI matrices [10]. Animal models deficient in collagen show signs of IVD degeneration, such as the acquisition of structural and functional matrix alterations [13,14,15,16]. These findings indicate an important association between MMPs and collagen homeostasis in IVD degeneration. IVD comprises many cell types, among which cells resembling chondrocytes (or chondrocyte-like cells with chondrocyte properties) in the nucleus pulposus and cartilaginous endplate of the IVD are most likely related to the regulation of the extracellular matrix. Similar to other chondrocytes in the body, the resembling chondrocytes also express MMPs [17] and play a key role in IVD degeneration [12]. Therefore, these cells have been used as targets for treating IVD degeneration in many studies [18,19].

Previous studies on twins strongly indicated that the genetic component is a predominant risk factor that contributes up to 74% heritability of lumbar spine etiology [20,21]. In recent years, several susceptibility genes associated with lumbar disk degeneration (LDD) have been identified in affected sib-pair linkage and candidate gene association studies [22,23]. Interestingly, several genes are related to the structure of IVD (such as *AGC*, *COL1A1*, *COL11A1*, *COL11A2*, *COL9A2*, and *COL9A3*), turnover of the extracellular matrix (such as *MMP-3*, *IL-1*, *IL-6*, *ADAMTS5*, *THBS2*, and *CHST3*), and connective tissues of the bone (such as *VDR* and *KIAA*). In addition, variants, such as IVS6 (-4) A > T of COL11A2 [24] and tryptophan alleles in COL9A2 (Trp2) [25] and COL9A3 (Trp3) [26], are associated with LDD. To the best of our knowledge, most genetic studies on degenerative LSS have focused on identifying susceptibility genes or variants in sporadic cases among the general population. Although most of the reported cases are sporadic, some familial cases of LSS have also been reported (OMIM: 152550), which were not associated with congenital skeletal dysplasia [27]. These findings suggest the involvement of unknown familial and degenerative mutations.

In this study, we aimed to identify the familial genetic cause of the disease through whole-exome sequencing (WES) analysis of a family with LSS. Seven family members had LBP, among whom four developed lumbar stenosis. Three of the four members with LSS had undergone spinal surgery and five were unaffected. We identified a novel mutation, c.417C > G (NM_002183, p.Y139X), in *IL3RA*, which was the only nonsense mutation identified using WES and was shared by all patients in the family. Subsequently, we examined the expression of IL3RA in human IVD and chondrocytes using immunofluorescence (IF) and investigated the regulatory roles of *IL3RA* on the expression of MMP and collagen genes through knockdown experiments in human chondrocytes. Our findings reveal a novel genetic cause of familial lumbar stenosis and demonstrate the potential pathogenic role of *IL3RA* in the disease.

## 2. Results

### 2.1. Clinical Features of the Family with Lumbar Stenosis

**Figure 1:**
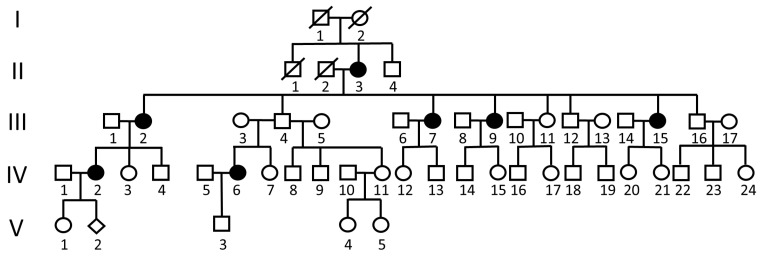
The pedigree of the family with low back pain and lumbar stenosis. The dark circles represent the patients with low back pain. Among them, II-3, III-2, III-7, and III-9 were diagnosed with lumbar stenosis and/or underwent spinal surgery.

A family across five generations participated in this study (Figure 1). Seven members (II-3, III-2, III-7, III-9, III-15, IV-2, and IV-6) had symptoms of LBP and/or leg weakness (Table 1). Four of them (II-3, III-2, III-7, and III-9) were clinically diagnosed with lumbar stenosis (Table 1). Patient II-3 developed LBP after prolonged standing or walking at 75 years of age. Patient III-2 underwent the first spinal surgery at 56 years of age due to severe LBP, soreness, numbness on the right side of the leg, and claudication. She underwent a second surgery at 63 years of age. Patient III-7 underwent spinal surgery at 51 years of age for lumbar stenosis and severe LBP. Postoperatively, the symptoms were relieved. However, her condition deteriorated progressively. She experienced back pain after standing and sitting for some time. Patient III-9 experienced occasional back pain after the age of 35 years and underwent surgery for acute disk herniation at 47 years. At 48 years of age, she underwent a second spinal surgery. Patient III-15 experienced recurrent LBP during her 20s. Her pain persisted, but the magnetic resonance imaging (MRI) findings at the age of 38 years were normal. The pain became severe after 53 years of age, making it difficult for her to sleep. However, radiographic examination did not reveal any spinal stenosis. Patient IV-2 was diagnosed with a herniated disk and bone spurs at 28 years of age. This condition was related to a weight gain of >30 kg after giving birth to her first child. Patient IV-6 experienced LBP and leg numbness at 45 years of age.

### 2.2. Filtering of the Candidate Mutations in the Family

To identify the underlying genetic cause of LDD in this family, we performed WES analysis for four family members with LBP and lumbar stenosis (II-3, III-2, III-7, and III-9), three family members with LBP only (III-15, IV-2, and IV-6), and five unaffected family members (II-1, II-4, III-4, III-12, and III-16). We identified 4545 heterozygous variants (SNVs and Indels) shared by the four patients. Among these, 182 variants were not detected in the five unaffected members. Only 5 of the 182 variants were nonsynonymous SNVs, including rs145576551, rs62250110, rs34552277, rs146883683, and rs564951643. However, these variants are common SNVs in East Asian populations, with a minor allele frequency (MAF) ranging from 0.14 to 0.48, according to the ExAC database. These MAFs are higher than the prevalence of LSS in the general East Asian population (11%, *n* = 6108, mean age 62 years, age range 19–93, and 56% female) [4]. Therefore, these SNVs were unlikely to be the major genetic variants causing familial LSS. If these variants were not causative of LSS in the family, no perfect segregation variant with the disease was identified in the analysis. There are several possible explanations for these results. First, unaffected family members did not serve as true healthy controls. They may develop the disease in the future but do not exhibit symptoms currently. Second, all affected members of this family (with LBP and/or LSS) were females, suggesting that LSS in this family could be caused by factors specific to females. For these reasons, we conducted another analysis of affected family members using WES data (Supplementary Materials, Table S1). All patients exhibited LBP symptoms before LSS diagnosis. LBP serves as an important warning sign of LSS, even in the absence of a clinical diagnosis. Therefore, we included three additional family members with LBP (III-15, IV-2, and IV-6) in the analysis in addition to the four LSS family members. A total of 587 variants were shared by the four members with LSS and three with LBP. After removing synonymous variants, 118 variants were identified. We then filtered out common variants with an MAF greater than 0.01 in East Asian populations (ExAC database). Eleven rare variants were identified. One of these, a nonsense mutation in *IL3RA* (NM_002183, c.417C > G, p.Y139X), drew our attention. This nonsense mutation is the only one among the 11 variants that causes deleterious effects. It is a novel mutation that has never been reported. We further investigated the potential role of the *IL3RA* nonsense mutation in this family.

### 2.3. Sequencing Validation of the Candidate Mutation

To verify the WES findings, we performed Sanger sequencing of *IL3RA* from all 12 family members (Supplementary Materials, Figure S1). Four LSS, three LBP, and three unaffected family members (II-4, III-4, and III-16; all males) had the mutation and were all heterogeneous. Two unaffected family members (II-1 and III-12) did not harbor this mutation. This result excludes the possibility that this mutation is a sex-linked inheritance of LSS. However, it cannot be ruled out that this mutation may promote the occurrence of LSS, along with other female-related factors, such as female hormones. Alternatively, there may be protective factors in males such as male hormones that offset or delay disease onset. To further investigate and validate our hypothesis, we conducted stepwise investigations and experiments.

### 2.4. Expression of IL3RA in Human IVD and Chondrocytes

**Figure 2:**
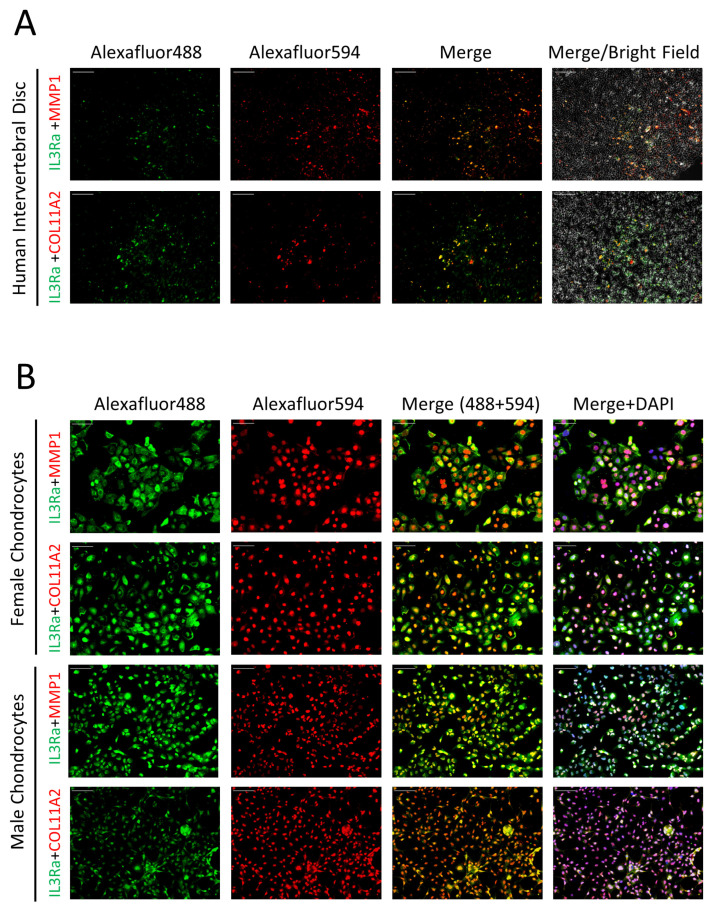
The expression of IL3RA (green), MMP-1 (red) COL11A2 (red), and DAPI (blue) in human intervertebral disk (**A**) and human chondrocyte cells (**B**). Bar = 20 μm.

To understand the contribution of IL3RA mutations to the LSS in the family, we investigated whether IL3RA is expressed in the IVD. As human IVD comprises cells with chondrocyte properties, we used chondrocyte-specific markers, MMP1 and COL11A2, to investigate whether IL3RA is expressed in chondrocytes or chondrocyte-like cells. Immunofluorescence (IF) staining showed that IL3RA was indeed expressed in human IVD and colocalized with chondrocyte-specific markers (Figure 2A). This suggests that IL3RA plays a role in cells with chondrocyte properties in human IVD. IF staining also revealed IL3RA expression in chondrocytes from females and males (Figure 2B).

### 2.5. Knockdown of IL3RA Affects the Total Amount of Collagen in Chondrocytes from Human Females and Males

**Figure 3:**
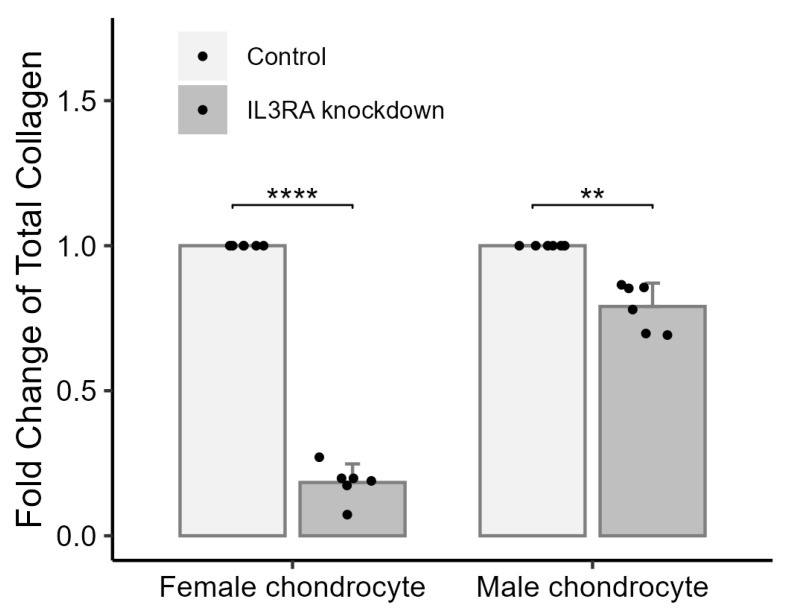
The effects of *IL3RA* knockdown on the total amount of collagen in chondrocyte lines from females and males. The difference between the *IL3RA* knockdown and control groups is presented as fold change. For each group, six individual experiments were performed. *n* = 6 each group. ** *p* < 0.01, **** *p* < 0.0001. Independent *t*-test was used for statistical analysis. The values are shown as mean with a 95% confidence interval (CI).

To elucidate the possible deleterious effects of the nonsense mutation in IL3RA in cells with chondrocyte properties in human IVD, we knocked down *IL3RA* in chondrocytes from females and males (C28/I2 and C20A4). In chondrocytes with *IL3RA* knockdown, the total collagen was 0.184-fold of that in the control group in chondrocytes from females and 0.791-fold of that in the control group in males (Figure 3). This result showed that *IL3RA* knockdown exerted a less deleterious effect on the total collagen content in male chondrocytes when compared with that in female chondrocytes.

### 2.6. Knockdown of IL3RA Affects the mRNA Expression of MMP1, MMP3, MMP10, and COL11A2 in Human Chondrocytes

**Figure 4:**
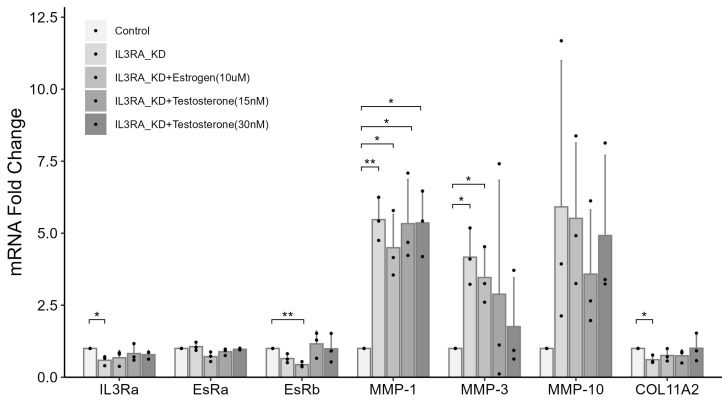
The effects of *IL3RA* knockdown on mRNA expression of estrogen receptors (*EsRa* and *EsRb*), *MMPs*, and *COL11A2* in chondrocytes from human females. The fold change in gene expression in each knockdown (KD) group compared with that in the non-KD control was determined using qPCR. For each group, the assay was performed in triplicate, and chondrocytes were treated with 10 nM of IL-3. The effects are presented for different concentrations of estrogen or testosterone. *n* = 3 each group. * *p* < 0.05, ** *p* < 0.01. Independent *t*-test was used for statistical analysis. The values are shown as mean with a 95% confidence interval (CI).

**Figure 5:**
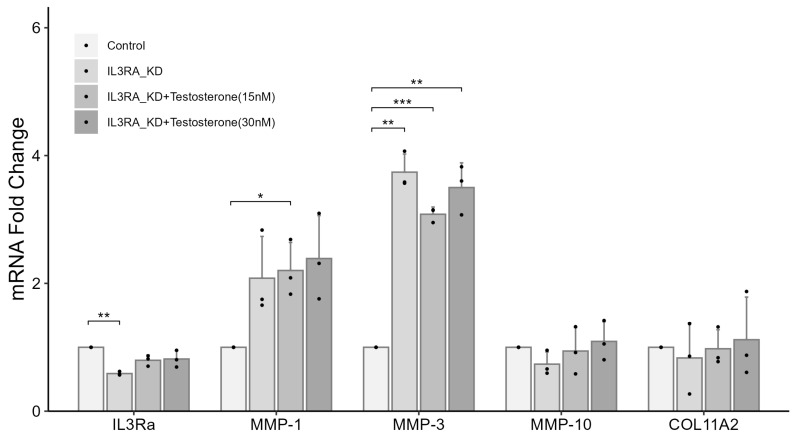
The effects of *IL3RA* knockdown on mRNA expression of *MMPs* and *COL11A2* in chondrocytes from human males. Fold change in gene expression in each knockdown (KD) group compared with that in the non-KD control was determined using qPCR. For each group, the assay was performed in triplicate, and chondrocytes were treated with 10 nM of IL-3. The effects are presented for different concentrations of testosterone groups. *n* = 3 each group. * *p* < 0.05, ** *p* < 0.01, and *** *p* < 0.001. Independent *t*-test was used for statistical analysis. The values are shown as mean with a 95% confidence interval (CI).

To understand the role of the nonsense mutation in IL3RA identified in chondrocytes from the family, we knocked down *IL3RA*. We also treated the cells with or without estrogen or testosterone to simulate the state before and after menopause. In chondrocytes from females, *IL3RA* mRNA level after knockdown was 0.589-fold of that in the control group. Compared with the control group, the mRNA levels of *MMP-1*, *-3*, and *-10* increased by 5.47-, 4.17-, and 5.91 times, respectively, whereas the mRNA level of *COL11A2* was 0.61-fold of that in the control group (Figure 4). In male chondrocytes, *IL3RA* mRNA level after knockdown was 0.589-fold of that in the control group. Compared with the control group, the mRNA levels of *MMP-1* and *-3* increased by 2.08 and 3.74 times, respectively (Figure 5). In addition, the effects of *IL3RA* knockdown on the mRNA expression of *MMPs* and *COL11A2* were not significantly reversed in chondrocytes from either females or males after treatment with 15 or 30 nM of testosterone (Figure 4 and Figure 5). These results showed that IL3RA deficiency affected the expression of *MMPs* and collagen genes in human chondrocytes. However, there were differences between females and males. The expression of *MMP-1* and *-3* increased in chondrocytes from both females and males, but the increase in mRNA expression was significantly higher in females. Additionally, the effects of *MMP-10* (higher expression) and *COL11A2* (lower expression) were observed only in female chondrocytes.

### 2.7. IL3RA Knockdown Reduces the Expression of EsRb and Affects the Expression of MMP-1, MMP-10, and COL11A2 in Female Chondrocytes

**Figure 6:**
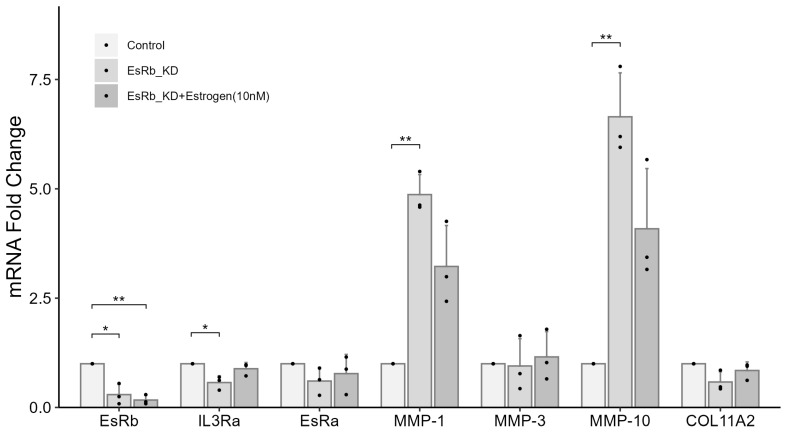
The effects of estrogen receptor beta (*EsRb*) gene knockdown on mRNA expression of *IL3RA*, estrogen receptor alpha (*EsRa*), *MMPs*, and *COL11A2* in chondrocytes from human females. Fold change in gene expression in each knockdown (KD) group compared with that in the non-KD control was determined using qPCR. For each group, the assay was performed in triplicate, and chondrocytes were treated with 10 nM of IL-3. The effects are presented for the 10 nM estrogen groups. *n* = 3 each group. * *p* < 0.05, ** *p* < 0.01. Independent *t*-test was used for statistical analysis. The values are shown as mean with a 95% confidence interval (CI).

In the experiment using female chondrocytes, the mRNA expression of estrogen receptor beta (EsRb) in the groups treated without or with 10 nM of estrogen after *IL3RA* knockdown was 0.649- and 0.439-fold of that in the control group, respectively (Figure 4). These results highlight the role of EsRb in regulating IL3RA, MMPs, and COL11A2. We investigated the effect of *EsRb* knockdown in chondrocytes from females. The mRNA expression of *EsRb* in the groups treated without or with 10 nM of estrogen after *EsRb* knockdown was 0.294- and 0.17-fold of that in the control group, respectively (Figure 6). In the group without estrogen treatment, the mRNA expression of *MMP-1* and *-10* increased by 4.87 and 6.65 times, whereas the mRNA expression of *COL11A2* and *IL3RA* was 0.583- and 0.57-fold of that in the control group, respectively. In contrast, in the group treated with 10 nM of estrogen, the mRNA expression of *MMP-1* and *-10* increased by 3.23 and 4.09 times, respectively. These results suggest that EsRb is involved in the regulation of MMPs and COL11A2 by IL3RA.

## 3. Discussion

LSS is one of the most common degenerative diseases that severely affects quality of life by causing pain and immobility. Recent studies have shed light on the genetic causes of this disease and the potential pathogenic role of IVD degeneration [22,23,24,25,26]. However, there are numerous cases of familial spinal stenosis of unknown etiology. In this study, we conducted a genetic analysis on a family afflicted with LSS to identify the mutation contributing to the disease. We identified a candidate mutation, c.417C > G (NM_002183, p.Y139X), located in IL3RA. *IL3RA* is located in the pseudoautosomal region (PAR1) of the X and Y chromosomes. All the genes characterized within PAR1 escape X inactivation [28]. Therefore, *IL3RA* is expected to exhibit an autosomal rather than a sex-linked inheritance pattern. We suspected that the mutation in *IL3RA* was the main cause of spinal disease in this family for various reasons. First, it was one of the few nonsynonymous mutations shared by all patients with LBP and/or LSS in the family. Second, it was the only nonsense mutation among the shared mutations that is expected to exert a considerable impact on the normal function of the protein owing to a premature stop codon. Third, the IL3 signaling pathway plays an important role in the physiological and pathological mechanisms of bone.

There is no direct evidence that IL3RA contributes to spinal diseases. However, IL3, a specific ligand of IL3RA, plays a chondroprotective role in osteoarthritis (OA), a degenerative disease of joints [29]. IL-3 reduced cartilage damage primarily by downregulating MMP expression and subsequently causing matrix degradation in vivo in a mouse model of human OA, and in vitro in mouse and human chondrocytes [29]. Importantly, chondrocytes can be regulated by IL3 because they express IL3RA. Joint degeneration, one of the main causes of IVD, may also involve the activation of MMPs and degradation of the matrix [30]. Based on the abovementioned evidence, we speculate that the IL3 pathway plays a similar chondroprotective role in spine health. Defects and/or decreases in the expression of IL3RA likely promote IVD degeneration and subsequent LDD by upregulating MMP expression and matrix degradation.

Our results firmly support our hypothesis. First, IL3RA is expressed in human IVD cells with chondrocyte properties and in human chondrocyte cell lines. Second, our results demonstrate that IL3RA plays an important role in the maintenance and stability of collagen in chondrocytes, especially in women. We further demonstrated that this effect might be mediated through the regulation of *MMP-1*, *MMP-3*, *MMP-10*, and *COL11A2* expression. MMPs may be crucial as their upregulation was greater than that of *COL11A2*. Importantly, the upregulation of these *MMPs* has been reported in human degenerated IVDs [12]. Therefore, defects or deficiencies in IL3RA likely promote IVD degeneration, resulting in LSS. In addition, our results indicate that the impact (fold change in upregulation) of a similar (approximately 41.1%) reduction in IL3RA expression on MMP-1 and -10 expression in chondrocytes was significantly greater in females than in males. This may also be one of the main reasons why *IL3R*A knockdown caused a differential reduction in total collagen content between males and females.

Furthermore, *IL3RA* knockdown led to a decrease in *EsRb* expression. *EsRb* knockdown resulted in an increase in the expression of *MMP-1* and *-10* and a decrease in the expression of *COL11A2* and *IL3RA*. These results suggest that EsRb is involved in the regulation of MMP-1, MMP-10, and COL11A2 by IL3RA. It also indicates that IL3RA and EsRb might mutually regulate each other. The addition of estrogen in each knockdown experiment reduced the expression of *MMP-1* and *-10* when compared with that in the absence of estrogen. We did not observe a similar effect in groups treated with testosterone. This indicates that testosterone may not have an effect on MMPs similar to that of estrogen. This suggests that estrogen and its receptor play a protective role in the loss of bone collagen by limiting the excessive activation of MMPs. Previous studies have demonstrated the important role of estrogen and MMPs in osteoimmunological diseases, such as OA, arthritis, and osteoporosis in women after menopause [31,32,33]. Regulating hormones and/or MMPs become potential therapeutic strategies [34,35,36]. Our results demonstrate the correlation between IL3RA and *EsRb* in regulating MMPs. This evidence suggests that IL3RA can become a novel treatment target for these diseases.

This study has several limitations. Although our study provided in vitro evidence of IL3RA function, whether the same cascade of inflammatory mediators and the protective effects of IL3RA on collagen content are present in different forms of disc degeneration in vivo requires further investigation. Since the spine surgeries were performed before this study, we could not obtain IVDs of those LSS patients. Currently, the role of IL3RA in the disease has not been fully examined in animal models and needs to be elucidated in IVDs of patients or IL3RA-knockout animals in future studies.

In conclusion, our results illustrate, for the first time, the association between IL3RA and EsRb and their importance and impact on MMPs and collagen in women. They also provide a possible explanation as to why only females in this family with the nonsense IL3RA mutation were affected. Along with the findings of previous studies, our results provide important evidence for the role of IL3 and IL3RA in the progression of bone and cartilage diseases, and for their use as potential therapeutic targets.

## 4. Materials and Methods

### 4.1. Sample Collection

Seven individuals diagnosed with LBP and/or LSS and five unaffected family members were recruited from Chi Hsien Spine Hospital (Taiwan). This study was approved by the Institutional Review Board of Academia Sinica of Taiwan (IRB No. AS-IRB01-18057). Informed consent was obtained from all the participants. LSS was diagnosed in all patients by Dr. Shiuh-Lin Hwang at the Chi Hsien Spine Hospital. Genomic DNA was extracted from whole blood using the Gentra Puregene Blood Kit (QIAGEN, Hilden, Germany), according to the manufacturer’s protocol. The purity and integrity of the extracted genomic DNA were verified using a NanoDrop 2000 spectrophotometer (Thermo Fisher Scientific, Sunnyvale, CA, USA) and DNA gel electrophoresis.

### 4.2. Exome Sequencing

The genomes of the four patients (II-1, II-4, III-4, III-12, and III-16) were sequenced and analyzed at the National Center for Genome Medicine (NCGM, Taipei, Taiwan). Genomic DNA, 200–300 bp in size, was fragmented using the Covaris S2 System (Covaris, Inc., Woburn, MA, USA) and purified using AMPure XP Beads (Agencourt Beckman Coulter, Beverly, MA, USA). The size distribution of the DNA fragments was evaluated with a High Sensitivity DNA Kit (Agilent Technologies, Palo Alto, CA, USA) on an Agilent BioAnalyzer 2100 system. The exome library was prepared using TruSeq™ DNA Library Preparation Kits v2 (Illumina, San Diego, CA, USA), following the manufacturer’s protocol. The size distribution of the exon library was evaluated using an Agilent BioAnalyzer 2100 system. Libraries were quantified using the 7900HT Fast Real-Time PCR System (Applied Biosystems, Foster City, CA, USA). Sequencing was performed with 101 bp paired-end reads on a HiSeq2000 (Illumina). We used a pipeline for NGS data analysis. Sequence alignment was conducted using bwa-mem (version 0.7.17-r1198-dirty) with an alt-aware strategy from the bwa.kit, with reference to the hs38DH sequence. Following alignment, duplicate reads were removed using samblaster, version 0.1.26. Alignment sorting was performed using Sambamba v0.5.4. Variant calling was performed using DeepVariant, which generated gVCF files. These gVCF files were then used in the joint calling analysis, which was performed using GLnexus. Variant annotation was performed using an ANNOVAR.

### 4.3. Polymerase Chain Reaction and Sanger Sequencing

The mutation (NM_002183, c.C417G, p.Y139X) in *IL3RA*, identified using NGS in all the patients, was verified using Sanger sequencing of PCR-amplified products using specific primers (Forward 5′-TCGAGTTCTCTTTCATGTTTGTG-3′; Reverse 5′-GTTCCCTGAGCATCCGTTT-3′) and GenTaq DNA polymerase (GM008-5, GenMark Technology, Taipei, Taiwan). PCR products were amplified according to the following program: 95 °C for 5 min, 95 °C for 30 s, 65 °C for 30 s, and 72 °C for 25 s, followed by 19 cycles at decreasing annealing temperatures in decrements of 0.5 °C per cycle, then 30 s at 95 °C, 15 s at 56 °C, 25 s at 72 °C for 15 cycles, and a final extension at 72 °C for 1 min. The PCR products were purified using an Exo-CIP PCR cleanup kit (New England Biolabs, Beverly, MA, USA) and subjected to Sanger sequencing using the DNA sequencer ABI 3730 (Applied Biosystems).

### 4.4. Cell Culture

Chondrocyte cell lines from human females (C28/I2) and males (C20A4) were purchased from Sigma-Aldrich (Sigma Co., St Louis, MO, USA). The chondrocytes were maintained in 1:1 mixture of Dulbecco’s modified Eagle medium (DMEM) and F12 medium (both from GIBCO, Life Technologies Corp., Carlsbad, CA, USA) supplemented with 10% (*v*/*v*) fetal bovine serum (FBS), 50 μg/mL ascorbic acid (Cat. No. A8960, Sigma-Aldrich), and 50 μM alpha-tocopherol (Cat. No. T1157, Sigma-Aldrich) in a 5% CO_2_ atmosphere at 37 °C, for 5–7 days. The culture medium was changed every 2–3 d until the cells were confluent.

### 4.5. Immunofluorescence and Imaging

Chondrocyte cells were seeded in chamber slides (Millicell EZ SLIDE) and fixed with 4% paraformaldehyde in phosphate-buffered saline (PBS) at 25 °C for 10 min. Tissue sections of formalin-fixed and paraffin-embedded human intervertebral disks were purchased from a commercial source OriGene (Rockville, MD, USA). Tissue sections were deparaffinized and digested overnight with hyaluronidase at 37 °C in a wet chamber. Nonspecific binding was blocked using 5% normal donkey serum and the slides were stained with mouse monoclonal anti-IL3RA (Cat. No. GTX60786; GeneTex, Taiwan) and rabbit polyclonal anti-COL11A2 (Cat. No. ab196613, Abcam, Cambridge, UK) or rabbit polyclonal anti-MMP1 (Cat. No. GTX100534, GeneTex). The secondary antibodies were diluted with donkey anti-mouse IgG Alexa Fluor 488 (Jackson ImmunoResearch, West Grove, PA, USA) and donkey anti-rabbit IgG Alexa Fluor 594 (Jackson ImmunoResearch). The slides were mounted and covered with an anti-fade permanent hard aqueous mounting solution (Cat. No. 16001; HNG, Taiwan) and coverslip. Fluorescence images were acquired using a ZEISS AxioVert A1 microscope (Carl Zeiss, Germany).

### 4.6. Determination of Collagen Content

The total collagen in cells was hydrolyzed with 10 N NaOH for 1 h at 120 °C. The hydrolysates were neutralized with 10 N HCl. The total collagen content was measured using a total collagen assay kit (Cat. No. 702440; Cayman Chemical) by reading the absorbance at 560 nm with a spectrophotometer (Spectramax 190 Microplate Reader, Molecular Devices).

### 4.7. siRNA and Transfection

C28/I2 and C20A4 immortalized human chondrocytes were cultured in a 1:1 mixture of DMEM and F12 medium supplemented with 50 μg/mL ascorbic acid, 50 μM alpha-tocopherol, 100 mM sodium pyruvate (Cat. No. 03-042-1B, Biological Industries), and 0.1576 mg/100 mL cysteine (Cat. No. C-1276, Sigma-Aldrich) for 1 day before transfection. For transfection, chondrocytes were first digested for 2 h at 37 °C in a DMEM/F12 medium containing hyaluronidase. After digestion, the supernatant was discarded, and the cells were washed twice with PBS. Transfection was performed using the OnceFect Transfection Reagent (Cat. No. 22001, HNG, Taiwan), according to the manufacturer’s instructions. The ratio of OnceFect Transfection Reagent (µL) to the total transfection volume (µL) was 6:100. According to the manufacturer’s protocol, siRNA (ON-TARGET plus, Dharmacon) was diluted with the medium to 100 nM. The OnceFect transfection reagent was added directly into the medium containing the diluted siRNA and incubated for 15 min at 25 °C, and then the mixture was added to the cells. After 24 h of transfection, the medium was replaced with fresh medium, and the cells were incubated with 10 nM of IL-3 (Cat. No. PHC0031, GIBCO), 10 nM of β-estradiol (Cat. No. E8875, Sigma-Aldrich), and 15 or 30 nM of testosterone (Cat. No. 86500; Sigma-Aldrich). The cells were harvested 24 h after treatment for mRNA analysis using real-time quantitative PCR.

### 4.8. Real-Time PCR

Total RNA was isolated using the RNAspin Mini Kit (Cat. No. 25-0500-71, Amersham, UK). cDNA was prepared using an oligo (dT) 20 primer and SuperScript III reverse transcriptase (Life Technologies). Gene expression was determined using the qPCR Master Mix (Cat. No. QPD0505; HighQu, Germany) on a ViiA7 Real-Time PCR System (Life Technologies). The primer sets are provided in the Supplementary Materials, Table S2. All data were normalized to GAPDH expression and analyzed using the ΔΔCt method.

### 4.9. Statistical Analysis

All experimental groups of mRNA expression were standardized to each control group. Statistical analysis was conducted using R version 4.2.2 accessed on 29 November 2022 (https://www.R-project.org/). All the data in this experiment are expressed as mean ± standard deviation (X ± S). Independent *t*-test was used for all comparisons. The results are presented as mean with 95% confidence intervals. A *p*-value < 0.05 was considered statistically significant.

## Figures and Tables

**Table 1:** Clinical and genetic characteristics of seven individuals with LBP and/or LSS.

	II-3	III-2	III-7	III-9	III-15	IV-2	IV-6
Sex	Female	Female	Female	Female	Female	Female	Female
Birth year	1926	1946	1951	1954	1964	1977	1972
Clinical symptoms and management	Low back pain after prolonged standing or walking	Low back pain since her 20s; numbness in the right leg; limp while walking; prone to falls; spine surgery at 56 and 63 years of age	Low back pain with muscle weakness since her 20s; persistent pain even after surgery; sciatica	Occasional back pain and soreness began at 37 years of age; soreness and numbness in lower limb; surgery for herniated intervertebral disk at 47 and 48 years of age	Occasional low back pain since her 20s; symptoms worsened significantly after 53 years of age	Low back pain, herniated disk, and bone spurs at 28 years of age	Low back pain and leg numbness after 45 years of age
Diagnosis	LSS	LSS	LSS	LSS	-	-	-
Age at diagnosis (years)	76	56	50	47	-	-	-
Radiograph	Confirmed using X-ray and MRI	Confirmed using X-ray and MRI	Confirmed using X-ray and MRI	Confirmed using X-ray and MRI	MRI failed to indicate a spinal anomaly	MRI failed to indicate a spinal anomaly	MRI failed to indicate a spinal anomaly
*IL3RA* variant	c.417C > G (p.Y139X)	c.417C > G (p.Y139X)	c.417C > G (p.Y139X)	c.417C > G (p.Y139X)	c.417C > G (p.Y139X)	c.417C > G (p.Y139X)	c.417C > G (p.Y139X)

## Data Availability

The data supporting the findings of this study are available within the article or the Supplementary Materials.

## Supplementary Materials

The following supporting information can be downloaded at https://www.mdpi.com/article/10.3390/ijms252010915/s1.

## References

[1] Kwon J.W., Moon S.H., Park S.Y., Park S.J., Park S.R., Suk K.S., Kim H.S., Lee B.H. (2022). Lumbar Spinal Stenosis: Review Update 2022. Asian Spine J..

[2] Siebert E., Pruss H., Klingebiel R., Failli V., Einhaupl K.M., Schwab J.M. (2009). Lumbar spinal stenosis: Syndrome, diagnostics and treatment. Nat. Rev. Neurol..

[3] Konno S., Kikuchi S., Tanaka Y., Yamazaki K., Shimada Y., Takei H., Yokoyama T., Okada M., Kokubun S. (2007). A diagnostic support tool for lumbar spinal stenosis: A self-administered, self-reported history questionnaire. BMC Musculoskelet. Disord..

[4] Jensen R.K., Jensen T.S., Koes B., Hartvigsen J. (2020). Prevalence of lumbar spinal stenosis in general and clinical populations: A systematic review and meta-analysis. Eur. Spine J..

[5] Gagne A.R., Hasson S.M. (2010). Lumbar extension exercises in conjunction with mechanical traction for the management of a patient with a lumbar herniated disc. Physiother. Theory Pract..

[6] Hennemann S., de Abreu M.R. (2021). Degenerative Lumbar Spinal Stenosis. Rev. Bras. Ortop..

[7] Browner M.F., Smith W.W., Castelhano A.L. (1995). Matrilysin-inhibitor complexes: Common themes among metalloproteases. Biochemistry.

[8] Takahashi M., Haro H., Wakabayashi Y., Kawa-uchi T., Komori H., Shinomiya K. (2001). The association of degeneration of the intervertebral disc with 5a/6a polymorphism in the promoter of the human matrix metalloproteinase-3 gene. J. Bone Jt. Surg. Br..

[9] Eser B., Eser O., Yuksel Y., Aksit H., Karavelioglu E., Tosun M., Sekerci Z. (2016). Effects of MMP-1 and MMP-3 gene polymorphisms on gene expression and protein level in lumbar disc herniation. Genet. Mol. Res..

[10] Goupille P., Jayson M.I., Valat J.P., Freemont A.J. (1998). Matrix metalloproteinases: The clue to intervertebral disc degeneration?. Spine.

[11] Roberts S., Caterson B., Menage J., Evans E.H., Jaffray D.C., Eisenstein S.M. (2000). Matrix metalloproteinases and aggrecanase: Their role in disorders of the human intervertebral disc. Spine.

[12] Vo N.V., Hartman R.A., Yurube T., Jacobs L.J., Sowa G.A., Kang J.D. (2013). Expression and regulation of metalloproteinases and their inhibitors in intervertebral disc aging and degeneration. Spine J..

[13] Sahlman J., Inkinen R., Hirvonen T., Lammi M.J., Lammi P.E., Nieminen J., Lapvetelainen T., Prockop D.J., Arita M., Li S.W. (2001). Premature vertebral endplate ossification and mild disc degeneration in mice after inactivation of one allele belonging to the Col2a1 gene for Type II collagen. Spine.

[14] Sarver J.J., Elliott D.M. (2004). Altered disc mechanics in mice genetically engineered for reduced type I collagen. Spine.

[15] Boyd L.M., Richardson W.J., Allen K.D., Flahiff C., Jing L., Li Y., Chen J., Setton L.A. (2008). Early-onset degeneration of the intervertebral disc and vertebral end plate in mice deficient in type IX collagen. Arthritis Rheumatol..

[16] Kibble M.J., Domingos M., Hoyland J.A., Richardson S.M. (2022). Importance of Matrix Cues on Intervertebral Disc Development, Degeneration, and Regeneration. Int. J. Mol. Sci..

[17] Cui Y., Yu J., Urban J.P., Young D.A. (2010). Differential gene expression profiling of metalloproteinases and their inhibitors: A comparison between bovine intervertebral disc nucleus pulposus cells and articular chondrocytes. Spine.

[18] Wang G., Huang K., Dong Y., Chen S., Zhang J., Wang J., Xie Z., Lin X., Fang X., Fan S. (2018). Lycorine Suppresses Endplate-Chondrocyte Degeneration and Prevents Intervertebral Disc Degeneration by Inhibiting NF-κB Signalling Pathway. Cell. Physiol. Biochem..

[19] Sakai D., Andersson G.B. (2015). Stem cell therapy for intervertebral disc regeneration: Obstacles and solutions. Nat. Rev. Rheumatol..

[20] Sambrook P.N., MacGregor A.J., Spector T.D. (1999). Genetic influences on cervical and lumbar disc degeneration: A magnetic resonance imaging study in twins. Arthritis Rheumatol..

[21] Battie M.C., Videman T., Gibbons L.E., Fisher L.D., Manninen H., Gill K. (1995). 1995 Volvo Award in clinical sciences. Determinants of lumbar disc degeneration. A study relating lifetime exposures and magnetic resonance imaging findings in identical twins. Spine.

[22] Kawaguchi Y. (2018). Genetic background of degenerative disc disease in the lumbar spine. Spine Surg. Relat. Res..

[23] Jiang H., Yang Q., Jiang J., Zhan X., Xiao Z. (2017). Association between COL11A1 (rs1337185) and ADAMTS5 (rs162509) gene polymorphisms and lumbar spine pathologies in Chinese Han population: An observational study. BMJ Open.

[24] Maeda S., Ishidou Y., Koga H., Taketomi E., Ikari K., Komiya S., Takeda J., Sakou T., Inoue I. (2001). Functional impact of human collagen alpha2(XI) gene polymorphism in pathogenesis of ossification of the posterior longitudinal ligament of the spine. J. Bone Miner. Res..

[25] Annunen S., Paassilta P., Lohiniva J., Perälä M., Pihlajamaa T., Karppinen J., Tervonen O., Kröger H., Lähde S., Vanharanta H. (1999). An allele of COL9A2 associated with intervertebral disc disease. Science.

[26] Paassilta P., Lohiniva J., Göring H.H., Perälä M., Räinä S.S., Karppinen J., Hakala M., Palm T., Kröger H., Kaitila I. (2001). Identification of a novel common genetic risk factor for lumbar disk disease. JAMA.

[27] Postacchini F., Massobrio M., Ferro L. (1985). Familial lumbar stenosis. Case report of three siblings. J. Bone Jt. Surg. Am..

[28] Helena Mangs A., Morris B.J. (2007). The Human Pseudoautosomal Region (PAR): Origin, Function and Future. Curr. Genom..

[29] Kour S., Garimella M.G., Shiroor D.A., Mhaske S.T., Joshi S.R., Singh K., Pal S., Mittal M., Krishnan H.B., Chattopadhyay N. (2016). IL-3 Decreases Cartilage Degeneration by Downregulating Matrix Metalloproteinases and Reduces Joint Destruction in Osteoarthritic Mice. J. Immunol..

[30] Dowdell J., Erwin M., Choma T., Vaccaro A., Iatridis J., Cho S.K. (2017). Intervertebral Disk Degeneration and Repair. Neurosurgery.

[31] Jehan F., Zarka M., de la Houssaye G., Veziers J., Ostertag A., Cohen-Solal M., Geoffroy V. (2022). New insights into the role of matrix metalloproteinase 3 (MMP3) in bone. FASEB Bioadv..

[32] Roman-Blas J.A., Castañeda S., Largo R., Herrero-Beaumont G. (2009). Osteoarthritis associated with estrogen deficiency. Arthritis Res. Ther..

[33] Martín-Millán M., Castañeda S. (2013). Estrogens, osteoarthritis and inflammation. Jt. Bone Spine.

[34] Lee Y.J., Lee E.B., Kwon Y.E., Lee J.J., Cho W.S., Kim H.A., Song Y.W. (2003). Effect of estrogen on the expression of matrix metalloproteinase (MMP)-1, MMP-3, and MMP-13 and tissue inhibitor of metalloproternase-1 in osteoarthritis chondrocytes. Rheumatol. Int..

[35] Burrage P.S., Mix K.S., Brinckerhoff C.E. (2006). Matrix metalloproteinases: Role in arthritis. Front. Biosci..

[36] Mei Y., Williams J.S., Webb E.K., Shea A.K., MacDonald M.J., Al-Khazraji B.K. (2022). Roles of Hormone Replacement Therapy and Menopause on Osteoarthritis and Cardiovascular Disease Outcomes: A Narrative Review. Front. Rehabil. Sci..

