# Supplementary material for: Role of IL3RA in a Family with Lumbar Spinal Stenosis
Source: Int J Mol Sci. 2024 Oct 10;25(20):10915. doi: 10.3390/ijms252010915 (PMC11507247; doi:10.3390/ijms252010915)
Supplement: Supplementary file 1 [file ijms-25-10915-s001.zip › ijms-3208195-supplementary.pdf]

Supp. Table S1. Filtering steps of NGS

Filtering step	SNV + Indel
**Dominant variants**	
Heterozygous variants shared by four patients	4,545
Heterozygous variants shared by three LBP patients	587
Removal of synonymous SNVs	118
Allele frequency less than 0.01 in ExAC EAS	11
Frameshift, splice site, start-loss, stop-loss, start-gain, or stop-gain mutations	1

Supp. Figure S1.

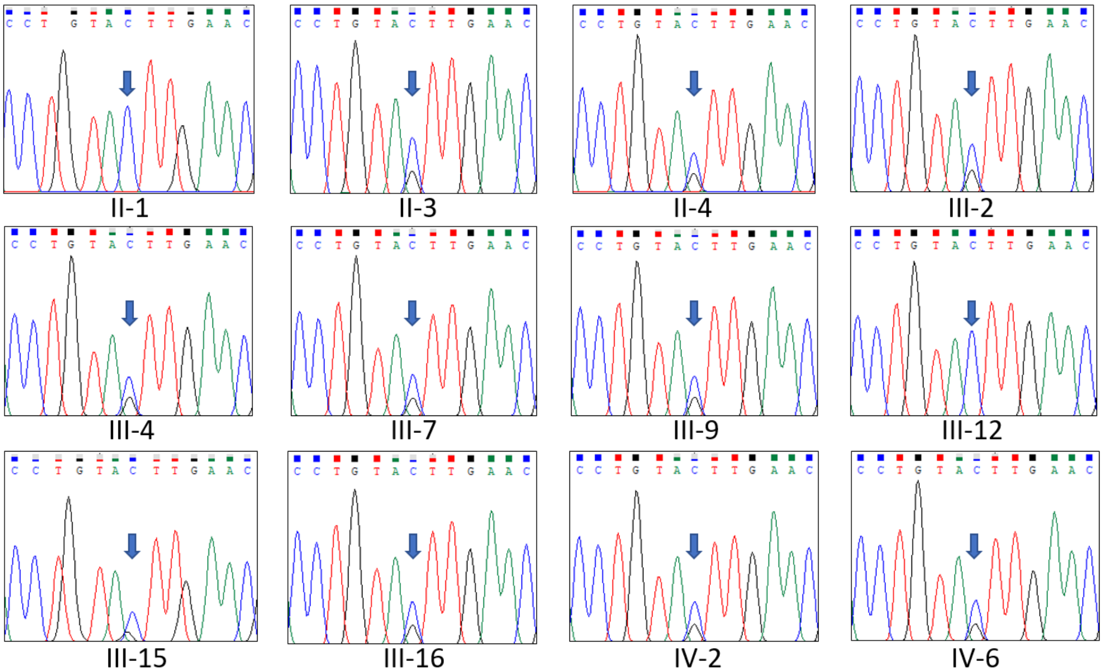

Figure S1. Sanger sequencing results of the c.417C>G mutation in *IL3Ra* in the family members with lumbar spinal stenosis. Mutation positions are indicated by arrows.

Supp. Table S2. Primers for real-time PCR

Gene		Sequence (5' to 3')	Length (bp)	Product size (bp)
IL3Ra	Forward	GGACGTCCAGTACGACCTGT	20	142
	Reverse	ACTTTGAGAACCGCTGGAGA	20	
EsRa	Forward	TGGGCTTACTGACCAACCTG	20	99
	Reverse	CCTGATCATGGAGGGTCAAA	20	
EsRb	Forward	AGAGTCCCTGGTGTGAAGCAA	21	143
	Reverse	GACAGCGCAGAAGTGAGCATC	21	
MMP-1	Forward	AGCTAGCTCAGGATGACATTGATG	24	58
	Reverse	GCCGATGGGCTGGACAG	17	
MMP-3	Forward	CACTCACAGACCTGACTCGG	20	81
	Reverse	AGTCAGGGGGAGGTCCATAG	20	
MMP-10	Forward	GACAGAAGATGCATCAGGCAC	21	133
	Reverse	GGCGAGCTCTGTGAATGAGT	21	
COL11A2	Forward	GCCTCAGCCTAGCAGAT	17	145
	Reverse	ATCACTCCATGGGTGTCCAATA	22	
GAPDH	Forward	GACCTGCCGTCTAGAAAAAC	20	126
	Reverse	TTGAAGTCAGAGGAGACCAC	20	
